# Cerebrospinal fluid markers including trefoil factor 3 are associated with neurodegeneration in amyloid-positive individuals

**DOI:** 10.1038/tp.2014.58

**Published:** 2014-07-29

**Authors:** R W Paterson, J W Bartlett, K Blennow, N C Fox, Alzheimer's Disease Neuroimaging Initiative, L M Shaw, J Q Trojanowski, H Zetterberg, J M Schott

**Affiliations:** 1 Department of Neurodegeneration, Dementia Research Centre, UCL Institute of Neurology, London, UK; 2 Department of Medical Statistics, London School of Hygiene and Tropical Medicine, London; 3 Department of Psychiatry and Neurochemistry, Institute of Neuroscience and Physiology, Sahlgrenska Academy, University of Gothenburg, Mölndal, Sweden; 4 Department of Pathology and Laboratory Medicine, Institute on Aging and Center for Neurodegenerative Disease Research, Perelman School of Medicine, University of Pennsylvania, Philadelphia, PA, USA; 5 Department of Molecular Neuroscience, UCL Institute of Neurology, London, UK

## Abstract

We aimed to identify cerebrospinal fluid (CSF) biomarkers associated with neurodegeneration in individuals with and without CSF evidence of Alzheimer pathology. We investigated 287 Alzheimer's Disease Neuroimaging Initiative (ADNI) subjects (age=74.9±6.9; 22/48/30% with Alzheimer's disease/mild cognitive impairment/controls) with CSF multiplex analyte data and serial volumetric MRI. We calculated brain and hippocampal atrophy rates, ventricular expansion and Mini Mental State Examination decline. We used false discovery rate corrected regression analyses to assess associations between CSF variables and atrophy rates in individuals with and without amyloid pathology, adjusting in stages for tau, baseline volume, p-tau, age, sex, ApoE4 status and diagnosis. Analytes showing statistically significant independent relationships were entered into reverse stepwise analyses. Adjusting for tau, baseline volume, p-tau, age, sex and ApoE4, 4/83 analytes were significantly independently associated with brain atrophy rate, 1/83 with ventricular expansion and 2/83 with hippocampal atrophy. The strongest CSF predictor for the three atrophy measures was low trefoil factor 3 (TFF3). High cystatin C (CysC) was associated with higher whole brain atrophy and hippocampal atrophy rates. Lower levels of vascular endothelial growth factor and chromogranin A (CrA) were associated with higher whole brain atrophy. In exploratory reverse stepwise analyses, lower TFF3 was associated with higher rates of whole brain, hippocampal atrophy and ventricular expansion. Lower levels of CrA were associated with higher whole brain atrophy rate. The relationship between low TFF3 and increased hippocampal atrophy rate remained after adjustment for diagnosis. We identified a series of CSF markers that are independently associated with rate of neurodegeneration in amyloid-positive individuals. TFF3, a substrate for NOTCH processing may be an important biomarker of neurodegeneration across the Alzheimer spectrum.

## Introduction

Although Alzheimer's disease (AD) is a relentless progressive condition there is considerable variation in the rate of progression between individuals.^1^ Previous studies have suggested that atrophy rates may be affected by the age of onset,^2^ disease severity,^3^ by the concurrence of other pathologies including vascular disease^4^ and TDP43 burden.^5^ However, the majority of the variance in rates of atrophy between individuals remains unaccounted for.^6^ A more detailed understanding of factors influencing this variability could allow for prognostication for patients, and aid in clinical trial design or interpretation where interindividual variance in atrophy rate increases required sample sizes;^6^ and provide insights into the underlying biology of AD, in turn leading to the discovery of new targets for disease prevention strategies. Biomarkers provide a means both of quantifying the rate of disease progression and exploring its influences. Rates of atrophy, either of brain or brain substructures can be measured with a high degree of precision from serially acquired MRI and provide a robust measure of progression which correlates with cognitive decline.^7^ Cerebrospinal fluid (CSF) can be used to assess neuronal, synaptic, inflammatory, and other proteins involved, or potentially involved, in AD pathogenesis.^8^ To date, Aβ1-42 and tau are routinely measured as AD biomarkers,^8^ with good evidence that these are markers of AD pathology and predict cognitive decline in mild cognitive impairment.^9^ What is less clear however is which CSF markers best reflect rates of neuronal damage or loss in AD—and therefore may be useful predictors of progression. A previous exploratory pilot study of CSF biomarkers in healthy elderly with amyloid pathology identified a number of analytes that may predict atrophy in specific brain regions.^10^ In this study we aimed to assess whether any analytes in a large panel of CSF biomarkers were associated with increased rates of atrophy across the Alzheimer spectrum.

## Patients and Methods

### Subjects

We investigated subjects from the Alzheimer's Disease Neuroimaging Initiative (ADNI) (adni.loni.ucla.edu), a multicenter publicly/privately funded longitudinal study of individuals with AD, amnestic mild cognitive impairment (MCI) and normal cognition. Institutional review boards approved the study and subjects gave written consent. Subjects underwent baseline and periodic clinical and neuropsychological assessment and serial MRI. Approximately 60% had CSF. A selected group had additional CSF analysis for the ADNI Biomarkers Consortium project ‘Use of Targeted Multiplex Proteomic Strategies to Identify Novel Cerebrospinal Fluid (CSF) Biomarkers in AD' as described on the ADNI website. We downloaded data from LONI (http://adni.loni.ucla.edu) that included all subjects with this supplementary CSF multiplex data. As our aim was to explore factors influencing atrophy rates in individuals with AD pathology we dichotomised subjects using a baseline CSF Aβ1-42 level of 192 pg ml^−1^, a level shown to distinguish individuals with autopsy confirmed AD pathology and controls with ~96% sensitivity and ~77% specificity.^11^ We excluded subjects who did not have useable 1.5T MRI scans at baseline and 1 year, and one subject without a defined diagnosis. We recorded ApoE status and genotype at the rs7280100 SNP, which has been identified a candidate locus for trefoil factor 3 (TFF3),^12^ as well as Mini Mental State Examination (MMSE) at baseline and 12 months.

### Cerebrospinal fluid

CSF collection, processing and storage procedures have previously been described.^11^ Processing, aliquoting and storage was carried out according to the ADNI Biomarker Core Laboratory Standard Operating Procedures (http://adni-info.org/Scientists/Pdfs/adniproceduresmanual12.pdf). Samples were analyzed using a multiplex-based immunoassay panel based upon Luminex immunoassay technology developed by Rules Based Medicine (MyriadRBM, Austin, TX, USA). CSF Aβ1-42, total tau (t-tau) and phosphorylated tau (p-tau) and a panel of 159 analytes including inflammatory, metabolic, lipid and other disease relevant analytes were tested. Data were prepared for analysis according to the biomarkers consortium statistical analysis plan (http://adni.loni.ucla.edu/wp-content/uploads/2012/01/2011Dec28-Biomarkers-Consortium-Data-Primer-FINAL1.pdf), and as previously described.^13^ Of 159 analytes, 76 had greater than 10% of quality control data missing, leaving 83 available for analysis. For each analyte, the normality of data was assessed by the ADNI Biomarkers Consortium: non-normal data were transformed using the Box and Cox technique.^14^

### Image acquisition

Details of the MRI methodology have previously been described.^15^ T1 weighted, inversion-recovery prepared structural images were acquired at baseline and 12 months on 1.5T MRI units using standardized protocols. Corrections for distortion due to gradient nonlinearity and for image intensity non-uniformity and scalings were made based on phantom measures. Images underwent central quality control evaluation for protocol compliance and internal quality control at the Dementia Research Centre.

### Volume loss measurement

Image analysis was performed using in-house MIDAS software.^16^ Whole brain and lateral ventricles were delineated semi-automatically and hippocampal volumes were measured using the automated HMAPS method.^17^ Volume loss (ml) between scans was obtained using the boundary shift integral (BSI) following a 9-degrees-of-freedom registration and differential bias correction of the follow-up to baseline scans. For lateral ventricles and hippocampi, change over time was quantified using the ventricular (VBSI)^18^ and hippocampal BSI (HBSI)^17^ respectively. Rates of volume loss were annualized using the interscan interval.

### Statistical analysis

To assess the relationship between CSF analytes and rates of brain volume change, we fitted separate regression models for rates of ventricular expansion, brain and hippocampal atrophy for each CSF variable, including baseline volume (brain, ventricular and hippocampal volume, respectively) and tau as covariates in both the amyloid positive and negative groups. Subsequent analyses in the amyloid-positive group alone were repeated including age, gender, APOE4 status and phospho-tau (p-tau) as additional covariates and finally also adjusting for baseline diagnosis (AD, MCI, control). We used an implementation of the false discovery rate (FDR) procedure^19^ with control at the 5% level to correct for multiple comparisons, and report the adjusted regression coefficients between each atrophy rate and those CSF variables showing FDR significant relationships. We then performed three exploratory reverse stepwise regression analyses to identify combinations of CSF analytes independently predicting increased rates of change for each measure, using FDR significant CSF variables identified in the preceding step and the three sets of adjustment variables. In each stepwise analysis, the corresponding adjustment variables were forced to be included in regression models. Finally, we assessed whether CSF analytes were associated with cognitive function. We first established whether there was a decline in MMSE between baseline and 12 months that was significantly different from zero. We then determined if atrophy rates and decline in MMSE scores at 12 months were correlated using separate regression models for rates of ventricular expansion, brain and hippocampal atrophy. We then fitted separate regression models for annualized change in MMSE score and each CSF variable found to be (after FDR control) associated with at least one atrophy measure.

The FDR procedure used was derived assuming independence between test statistics.^19^ However, it has been shown that the procedure is valid under certain types of dependence,^20^ and in a simulation study (results not shown) matching the analysis used here, the procedure correctly controlled the FDR at 5%.

To quantify the unadjusted group discrimination ability of the analytes that were FDR significant adjusted for baseline brain volumes, sex, age, APOE4 status, tau and p-tau the area under the ROC curve for detecting between AD and control groups was estimated.

## Results

### Baseline group characteristics

**Figure 1:**
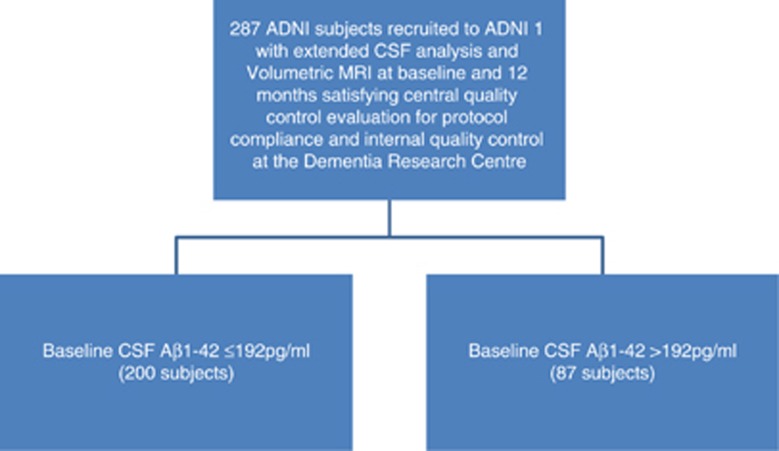
Subjects included in analysis. ADNI, Alzheimer's Disease Neuroimaging Initiative; CSF, cerebrospinal fluid.

A flow chart outlining subject inclusion is provided in Figure 1

The demographics, genetic characteristics, cognitive scores and atrophy measures of the 287 subjects included in this analysis are described in Table 1. The mean±s.d. age of this group was 74.9±6.9 years, 21.6% had a clinical diagnosis of AD, 48.1% MCI and 30.3% were controls. Eighty-three CSF analytes as well as CSF tau, Aβ1-42 and p-tau were available for analysis (Supplementary Table 1).

### Analytes predicting atrophy

In subjects without evidence for significant amyloid deposition, that is, those with baseline CSF Aβ1-42 >192 pg ml^−1^, after adjusting for baseline brain volumes and CSF t-tau and with FDR correction to control for multiple comparisons, none of the CSF analytes was significantly associated with any of the atrophy measures.

**Figure 2:**
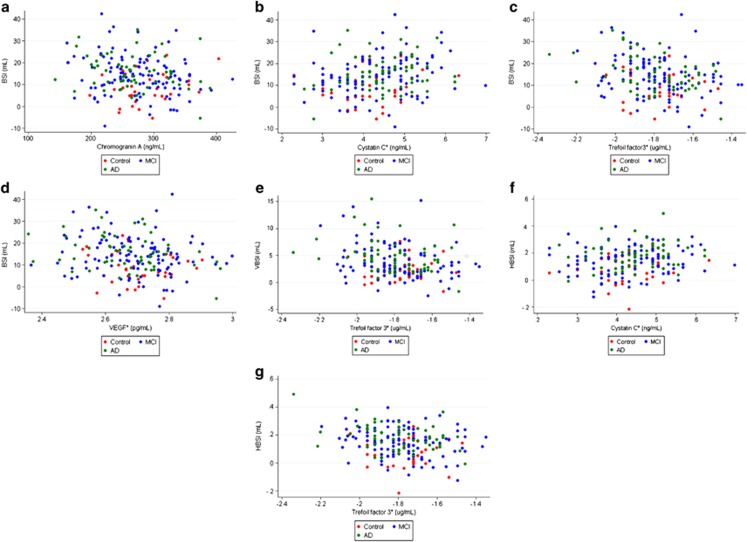
Scatter plots of annualized atrophy (BSI—whole brain atrophy, HBSI—hippocampal atrophy, VSBI—ventricular expansion) against analytes for those found to be associated (after FDR correction) with rates of volume change after adjusting for baseline volume, sex, age, APOE4 status, t-tau and p-tau. *, transformed data. Where data has been transformed (TFF3, CysC, VEGF), the units relate to data before transformation. AD, Alzheimer's disease; CSF, cerebrospinal fluid; BSI, boundary shift integral; FDR, false discovery rate; MCI, mild cognitive impairment; p-tau, phosphorylated tau; t-tau, total tau; TFF3, trefoil factor 3.

In subjects with CSF Aβ1-42 ⩽192 pg ml^−1^, after adjusting for baseline volumes and t-tau and with FDR correction to control for multiple comparisons, 10/83 analytes were associated with whole brain atrophy rate, 45/83 analytes with ventricular expansion rate, and 4/83 with hippocampal atrophy rate (Table 2). After additionally adjusting for p-tau, age, ApoE status and sex and with FDR correction to control for multiple comparisons, 4/83 analytes were associated with whole brain atrophy rate, 1/83 analyte with ventricular expansion rate, and 2/83 with hippocampal atrophy rate (Table 2). These relationships are illustrated using scatter plots in Figure 2. After additionally adjusting for baseline diagnosis, only 2/83 analytes were associated with hippocampal atrophy, and none with brain atrophy or ventricular expansion (Table 2).

Lower levels of TFF3 were consistently associated with greater ventricular expansion (*P*<0.001), hippocampal atrophy rate (*P*<0.001) and whole brain atrophy rate (*P*<0.001) even after adjusting for baseline brain volumes, t-tau, p-tau, age, APOE status and sex. After additionally adjusting for baseline diagnosis, lower levels of TFF3 were still associated with higher hippocampal atrophy (*P=*0.007). Higher levels of Cystatin C (CysC) were positively associated with all three atrophy measures after adjusting for baseline brain volumes and t-tau and remained predictive of higher whole brain (*P=*0.009) and hippocampal atrophy (*P=*0.034) after adjusting for p-tau, age, ApoE status and sex. Lower levels of vascular endothelial growth factor (VEGF) were positively associated with all three atrophy measures after adjusting for baseline brain volumes and t-tau and remained associated with higher whole brain atrophy (*P=*0.023) after adjusting for p-tau, age, APOE status and sex. Lower levels of Chromogranin-A (CgA) were associated with higher whole brain atrophy (0.008) and ventricular expansion (0.009) after adjusting for baseline volumes and t-tau and predicts higher whole brain atrophy (*P=*0.009) after additionally adjusting for p-tau, age, ApoE status and sex.

In exploratory reverse stepwise models that included only those variables showing FDR significant associations in the initial analyses (Table 3), lower levels of TFF3 were associated with higher rates of whole brain atrophy, ventricular expansion and hippocampal atrophy, even when, p-tau, age, APOE status and sex were included in the model. Lower levels of CgA were associated with higher whole brain atrophy and ventricular expansion when t-tau and baseline volume were included in the model; and with higher whole brain atrophy when p-tau, age, APOE status and sex were also included as covariates. In addition, adjusting for baseline diagnosis, TFF3 was the only analyte independently associated with hippocampal atrophy rate; and no analytes were (independently) associated with whole brain atrophy or ventricular expansion.

Twenty-three of the subjects had a minor allele at the rs7280100 locus (predicted to reduce CSF TFF3). These individuals had 18% higher rates of ventricular expansion, 14% higher rates of brain atrophy and 30% higher rates of hippocampal atrophy compared with noncarriers, the latter reaching borderline significance after adjustment for baseline diagnosis, (*P=*0.07).

### Analytes predicting cognitive decline

In subjects with CSF Aβ1-42 >192 pg ml^−1^, MMSE data at 12 months was available for all 87 subjects. The average decline was not statistically significantly different from zero and therefore no further regression analyses were conducted in this group.

In subjects with CSF Aβ1-42 ≤192 pg ml^−1^, MMSE data at 0 and 12 months was available for 199 subjects, who declined on average by 1.7±3.5 points per year. Change in MMSE score at 12 months was strongly associated with change in whole brain atrophy rate (regression coefficient=−0.15, *P*<0.001), ventricular expansion (−0.49, *P*<0.001), and hippocampal atrophy rate. (−11.16, *P*<0.001) Baseline levels of 11 CSF markers—AXL, ApoE, CD-40 antigen, CgA, cystatin C, M-CSF, matrix metalloproteinase-2 (MMP-2), pregnancy-associated plasma protein, tissue factor, TFF3 and VEGF—were significantly (without FDR correction) associated with decline in MMSE at 12 months.

### Predictive value

For those analytes that were FDR significant in Table 2, the area under the ROC curve for detecting between AD and control groups were 0.59 (95% confidence interval=0.50, 0.69; CgA), 0.55 (0.45, 0.64; CysC), 0.55 (0.45, 0.65; TFF3) and 0.61 (0.52, 0.70; VEGF). For reference, the corresponding estimated values for t-tau and p-tau were 0.85 and 0.83, respectively.

## Discussion

Current models of AD pathogenesis suggest that deposition of brain Aβ is a very early feature of AD, probably occurring before the onset of AD-related neuronal loss (that is, neurodegeneration).^21^ Rates of atrophy are significantly increased in individuals with established AD,^22^ mild cognitive impairment due to AD,^23^ and in asymptomatic brain amyloidosis,^24^ and correlate more closely with cognitive decline and disease progression than amyloid burden or rate of accumulation.^25^ In this study, using a panel of analytes selected on the basis of relevance to a range of different diseases including cancer and autoimmune disorders as well as AD, we have identified a number of CSF biomarkers associated with increased rates of neurodegeneration. In particular, our results suggest that in individuals with evidence for brain amyloid deposition, CSF TFF3 level is associated both with rate of cognitive decline and with rates of brain and hippocampal atrophy and ventricular expansion.

Whilst we failed to find an association between any analyte and rate of atrophy in the amyloid negative group, after allowance for multiple comparisons in the amyloid-positive group in which we adjusted for baseline brain volume and t-tau, we found that 45 analytes predicted increased ventricular expansion and ten predicted rate of whole brain atrophy. Ventricular expansion and brain atrophy are closely correlated,^26^ and as expected, all ten factors predicting increased rates of whole brain atrophy also predicted increased ventricular expansion. The higher precision with which rate of ventricular change can be quantified^18^ is likely to explain the larger number of analytes associated with ventricular expansion compared with whole brain loss.

When we additionally adjusted for p-tau, ApoE status and sex, adjusting for multiple comparisons, the number of factors associated with rates of neurodegeneration in the amyloid-positive group alone was considerably reduced, with only four analytes showing an association with rates of neurodegeneration. Lower levels of TFF3, VEGF and CrA and higher levels of CysC were associated with increased rates of brain atrophy; lower levels of TFF3 with increased ventricular expansion; and lower levels of TFF3 and higher levels of CysC with increased rates of hippocampal atrophy. In exploratory reverse stepwise analyses, TFF3 was significantly associated with rate of decline in all three measures (Table 3), with an effect both independent from and at least as great as CSF t-tau, which as expected, also (unadjusted) predicted all three measures of neurodegeneration.^27^ The effect of TFF3 persisted even once baseline p-tau, age, sex and APOE4 status had been accounted for (Table 3), and was still associated with rate of hippocampal atrophy even once clinical diagnosis (for example, control/MCI/AD) had been accounted for. These results therefore support an association between CSF TFF3 and increased rates of neurodegeneration independent of established CSF biomarkers in individuals with amyloid deposition, suggesting that CSF TFF3 may be a novel and valuable biomarker of decline across the spectrum of AD.

### Trefoil Factor 3 (TFF3)

Encoded by the TFF3 gene on chromosome 21, TFF3 is a protein expressed by secretory epithelial cells principally in the gastrointestinal tract, and also in human hypothalamus and pituitary,^28^ and in the hippocampi, temporal cortices and cerebellum of mice.^29^ Its function in the central nervous system is unknown,^29^ although TFF3 administration to mice has been reported to improve memory.^30^ In the periphery, TFF3 has important roles in NOTCH processing, and measurement of TFF3 in blood/urine/faeces has been patented^31^ and used^32^ as a means of assessing NOTCH-related side-effects in trials of gamma-secretase inhibitors for the treatment of AD. To our knowledge, this is the first study to implicate TFF3 as a marker for neurodegeneration across the AD spectrum, and furthermore to show that this is independent of t-tau and p-tau: while there are few data on which to suggest mechanisms, one intriguing possibility is that this effect might in some way be mediated by alterations in gamma-secretase processing. While numbers with a minor allele were too small for anything more than an exploratory analysis, the observation that genotype at the rs7280100, a candidate locus associated with TFF3, is intriguing, and if replicated in independent samples, suggests that CSF TFF3 and/or the rs7280100 genotype may both help predict the rate of neurodegeneration in individuals with amyloid pathology; and that elucidating the function of TFF3 in the central nervous system may provide insights into mechanisms influencing neurodegeneration in the presence of brain amyloidosis.

### Cystatin C (CysC), Vascular Endothelial Growth Factor (VEGF) and Chromogranin-A (CgA)

Of the other three biomarkers emerging prominently from our analyses, CysC colocalizes with β amyloid in amyloid plaques, amyloid-laden vascular walls in cerebral amyloid angiopathy and in Down's syndrome and is typically reduced in AD CSF, with multiple lines of evidence suggesting that it has protective roles in AD principally due to influences on amyloid processing and deposition.^33^ Conversely, increased CysC immunoreactivity is seen in specific neuronal population in AD suggesting a role in neurodegeneration;^33^ and in dopaminergic neurons, CysC has been shown to have a role in neuronal injury-mediated microglial activation and neurotoxicity.^34^ Our finding of a positive relationship between rates of atrophy and CysC, in individuals in whom amyloid deposition has already occurred, could therefore be explained in terms of a harmful neuroinflammatory response which results in neuronal damage. VEGF, abundantly expressed in the CNS, has roles in modulation of angiogenesis, vascular remodelling, repair, permeability and inflammation,^35^ and is involved in microglial chemotaxis perhaps reflecting an early response to amyloid deposition.^36^ Our finding of increased atrophy with lower levels of CSF VEGF is consistent with VEGF having a protective role in AD, and in keeping with reports that transgenic AD mice with increased neuronal expression of VEGF have a functional improvement in memory,^35^ suggests that upregulation of VEGF may be a useful therapeutic strategy for AD. Increased levels of CSF VEGF has been seen in individuals with AD and vascular dementia compared with controls^37^—this could also represent a protective response although VEGF levels were not correlated with rate of atrophy or rate of cognitive decline., The neuroendocrine secretory protein CgA is the major protein of large dense-core synaptic vesicles and may be a marker of synaptic dysfunction.^38^ In one study lower CSF levels of CgA were reported in the CSF of subjects with early onset sporadic or familial Alzheimer's disease,^39^ potentially in keeping with our finding of inverse relationship between CgA level and increased rates of brain atrophy and ventricular expansion.

In a previous study using this same panel of analytes, ten CSF measures (ACE, CgA, AXL, TNF-related apoptosis-inducing ligand receptor, CD-40, M-CSF, beta-2-microglobulin, stem cell factor, CLU and IL-3) were shown to predict increased rates of amyloid deposition in cognitively normal elderly individuals.^13^ When comparing these results aiming at identifying markers predicting rate of amyloid accumulation in healthy controls with ours (assessing rate of neurodegeneration in individuals with likely amyloid pathology) it is notable that in our initial analysis, we identified five CSF analytes common to both: AXL, CgA, CLU, IL-3 and M-CSF; and including more stringent covariates that CgA remained a consistent finding. While this could reflect that rates of amyloid deposition and brain atrophy are highly correlated—as would be predicted in the mid-phase of AD pathogenesis—this would also be consistent with common mechanisms linking amyloid deposition to subsequent neurodegeneration. However, the fact that TFF3, CysC and VEGF were not identified in previous analyses may suggest that these analytes may be exerting their effects on neurodegeneration independent of amyloid deposition.

This study has a number of limitations. The number of subjects is relatively small, particularly relative to the number of analytes, and these findings thus require replication in other, larger cohorts. However, we have used a statistical procedure to control for multiple comparisons, indicating that the evidence for associations is moderately strong. While the use of reverse stepwise analysis must be considered exploratory, the consistency with which TFF3 emerges as a strong independent predictor of atrophy is striking. However, it is perhaps less surprising that the same analytes often predict all three atrophy measures, given that the atrophy measures are mutually correlated. A relatively small percentage of the variance in atrophy rates is explained by these findings suggesting that other factors and other biomarkers reflecting other independent pathways have yet to be identified, noting that a certain proportion of variance may also be due to measurement error. Including all individuals with low Aβ1-42 in our analysis assumes that all patients with brain amyloidosis are on the same neuropathological spectrum. While larger, more homogeneous samples are required to assess whether the relationships we show are driven by individuals with asymptomatic amyloidosis, MCI or established AD, it is notable that the relationship between TFF3 and hippocampal atrophy remains even after adjusting for clinical diagnosis—which in this study probably simply reflects different stages of disease.

We have identified a number of CSF markers that may be associated with the rate of neurodegeneration in individuals with amyloid deposition. These candidate biomarkers warrant further investigation, potentially providing prognostic information for patients; covariates for clinical trials; and insights into AD biology. While several of the CSF biomarkers hint at immune-mediated links between responses to amyloid deposition and brain volume loss, the function of TFF3, which we found to be the single strongest predictor of neurodegeneration across the spectrum of brain amyloidosis, is unknown. Further studies to replicate these findings and in particular to investigate the role of TFF3 in the pathogenesis of AD are required.

## Figures and Tables

**Table 1:** Baseline demographics, ApoE genotype, cognitive profiles, CSF profiles, brain volumes and 1 year atrophy rates of 200 subjects with Aβ1-42≤192 pg ml^−1^ and 87 subjects with Aβ1-42>192 pg ml^−1^

	*Subjects with Aβ1-42* ≤*192 pg* *ml*^−1^ *(*n*=200)*			
	*Combined*	*Controls (*n*=33)*	*MCI (*n*=108)*	*AD (*n*=59)*
Age at CSF exam (years)	74.8±6.7	76.5±5.5	74.4±7.0	74.5±7.4
Gender (% male)	59.4	54.5	63.9	52.5
APOE4 positive (%)	65.5	51.5	69.0	78.0
MMSE (mean)	26.1±2.5	29.1±0.1	26.7±1.8	23.5±1.8
Modified ADAS-cog	13.1±6.2	7.1±3.4	12.1±4.6	18.5±6.1
Aβ1-42 (pg ml^−1^)	137.2±22.7	146.5±25.5	136.5±29.0	133.1±23.2
t-tau (pg ml^−1^)	114.4±55.3	82.5±30.7	115.3±56.0	130.7±57.7
p-tau (pg ml^−1^)	39.5±17.5	31.2±17.4	39.8 ±15.4	43.6±19.8
KBSI (ml per year)	13.8±8.7	8.9±7.3	14.2±9	15.7±7.8
VBSI (ml per year)	3.8±3.1	2.1±2.0	3.8±3.1	4.9±3.1
HBSI (ml per year)	0.15±0.10	0.08±0.1	0.1±0.1	0.2±0.1
MMSE decline from baseline to 12 months (points per year; *n*=199)	1.7±3.5	0.4±1.7	1.4±2.7	3.0±4.8
% of individuals with minor allele rs7280100	12.2	12.5	10.9	14.6

	*Subjects with Aβ1-42>192 pg* *ml*^−1^ *(*n*=87)*			
	*Combined*	*Controls (*n*=54)*	*MCI (*n*=30)*	*AD (*n*=3)*
Age at CSF exam (years)	75.4±6.3	74.9±5.2	75.5±8.0	81.4±6
Gender (% male)	62.1	50	80	100
APOE4 positive (%)	89.6	90.7	86.7	100.0
MMSE (mean)	28.3±1.7	29.0±1.0	27.2±1.9	25.3±1.2
Modified ADAS-cog	7.6±1.4	5.7±2.5	10.5±3.8	14.4±6.8
Aβ1-42 (pg ml^−1^)	244.4±26.1	243.1±25.6	246.5±26.0	247.0±45.7
t-tau (pg ml^−1^)	62.2±20.9	60.8±21.4	64.3±20.2	66±22.3
p-tau (pg ml^−1^)	21.0±8.0	20.6±7.9	21.8±8.5	21.3±7.5
KBSI (ml per year)	6.0±6.7	4.7±5.4	7.5±8.0	12.6±10.5
VBSI (ml per year)	1.4±1.6	1.1±1.1	1.7±2.1	2.5±2.5
HBSI (ml per year)	0.05±0.1	0.03±0.09	0.07±0.1	0.1±0.06
MMSE decline from baseline to 12 months (points per year; *n*=199)	0.1±1.6	0.06±1.3	0.2±2	0±2
% of individuals with minor allele rs7280100	12.0	13.2	11.1	0

Abbreviations: AD, Alzheimer's disease; ADAS-cog, Alzheimer's Disease Assessment Scale-cognitive subscale; CSF, cerebrospinal fluid; HBSI, hippocampal boundary shift integral; KBSI, whole brain boundary shift integral; MCI, mild cognitive impairment; MMSE, mini mental state examination; p-tau, phosphorylated tau; t-tau, total tau; VBSI, ventricular boundary shift integral. Mean±s.d. provided unless stated.

**Table 2:** Regression coefficients for dependence of atrophy measures on CSF with control for the false discovery rate in subjects with low CSF Aβ1-42 (⩽192 pg ml^−1^): adjusted for baseline brain volumes and tau; adjusted for baseline brain volumes, sex, age, APOE4 status, t-tau and p-tau; adjusted for baseline brain volumes, sex, age, APOE4 status, t-tau, p-tau and baseline diagnosis

	*Brain atrophy*		*Ventricular expansion*		*Hippocampal atrophy*	
	*Regression coefficient*	P	*Regression coefficient*	P	*Regression coefficient*	P
*Adjusting for baseline brain volumes and t-tau*						
Alpha-1-Microglobulin^a^ (μg ml^−1^)			−3.17	0.002		
Alpha-2-Macroglobulin^a^ (mg ml^−1^)			−4.31	0.006		
Alpha-1-Antitrypsin^a^ (mg ml^−1^)			−0.19	0.018		
Angiotensin-converting enzyme^a^ (ng ml^−1^)			−3.17	0.027		
Angiopoietin-2^a^ (ng ml^−1^)			−3.24	0.013		
Apolipoprotein A-I^a^ (mg ml^−1^)			−2.39	0.018		
Apolipoprotein C-III^a^(μg ml^−1^)			−2.02	0.042		
Apolipoprotein D^a^ (μg ml^−1^)			−3.54	0.003		
Apolipoprotein E^a^ (μg ml^−1^)	−14.71	0.017	−0.26	0.002		
Apolipoprotein H^a^ (μg ml^−1^)			−2.99	0.004		
AXL receptor tyrosine kinase (ng ml^−1^)	−1.59	0.01	−0.40	0.018		
Beta-2-Microglobulin^a^ (μg ml^−1^)			−3.87	0.018		
Complement C3^a^ (mg ml^−1^)			−3.80	0.003		
CD-40 antigen^a^ (ng ml^−1^)			−5.20	0.004		
Chromogranin-A (ng ml^−1^)	−0.05	0.008	−0.013	0.009		
Clusterin^a^ (μg ml^−1^)	−11.90	0.023	−4.20	0.002		
Cystatin-C^a^ (ng ml^−1^)	32.89	0.003	10.77	<0.001	0.34	0.019
Fibroblast growth factor 4^a^ (pg ml^−1^)			3.45	0.022		
Fibrinogen^a^ (mg ml^−1^)			−0.28	0.001		
Heparin-binding EGF-like growth factor^a^ (pg ml^−1^)			−4.08	0.048		
Hepatocyte growth factor^a^ (ng ml^−1^)			−2.59	0.048		
Immunoglobulin A^a^ (mg ml^−1^)			−1.28	0.048		
Interleukin-3^a^ (ng ml^−1^)	−5.66	0.036	−2.02	0.003		
Insulin-like growth factor-binding protein (ng ml^−1^)			−0.017	0.004		
Interferon gamma induced Protein 10^a^ (pg ml^−1^)			−0.17	0.034		
Lectin-like oxidized LDL receptor 1 (ng ml^−1^)			−0.30	0.006		
Macrophage colony-stimulating factor 1^a^ (ng ml^−1^)	−15.02	0.023	−5.89	<0.001		
Monokine induced by gamma interferon^a^ (pg ml^−1^)			−1.66	0.009		
Neutrophil gelatinase-associated lipocalin^a^ (ng ml^−1^)			−2.49	0.018		
N-terminal prohormone of brain natriuretic peptide^a^ (pg ml^−1^)			−3.91	0.006	−0.15	0.035
Placenta growth factor^a^ (pg ml^−1^)			−2.34	0.043		
Pancreatic polypeptide^a^ (pg ml^−1^)			−1.31	0.038		
Serum amyloid P-component^a^ (μg ml^−1^)			−1.56	0.03		
Stem cell factor^a^ (pg ml^−1^)			−0.18	0.027		
Sex hormone-binding globulin^a^ (nmol/l)			−2.23	0.018		
Thyroxine-binding globulin^a^ (μg ml^−1^)			−0.18	0.023		
Tissue factor^a^ (μg ml^−1^)	−11.79	0.017	−3.73	0.003		
Trefoil factor 3^a^ (μg ml^−1^)	−14.69	0.003	−6.19	<0.001	−0.178	0.002
Tissue inhibitor of metalloproteinases 1^a^ (ng ml^−1^)			−3.88	0.015		
Thrombomodulin^a^ (ng ml^−1^)			−2.52	0.045		
Tumor necrosis factor receptor 2^a^ (ng ml^−1^)			−4.05	0.007		
TNF-related apoptosis-inducing ligand receptor 3^a^ (ng ml^−1^)			−4.05	0.004		
Vascular cell adhesion molecule-1^a^ (ng ml^−1^)			3.46	0.018		
Vascular endothelial growth factor^a^ (pg ml^−1^)	−21.32	0.004	−6.86	<0.001	−0.21	0.034
von Willebrand factor^a^ (μg ml^−1^)			−4.18	0.003		
						
*Adjusting for baseline brain volumes, sex, age, APOE4 status, tau and p-tau*						
Chromogranin-A (ng ml^−1^)	−0.05	0.009				
Cystatin-C^a^ (ng ml^−1^)	32.58	0.009			0.35	0.034
Trefoil factor 3^a^ (μg ml^−1^)	−16.43	0.009	−4.46	0.03	−0.23	0.001
Vascular endothelial growth factor^a^ (pg ml^−1^)	−20.09	0.023				
						
*Adjusting for baseline brain volumes, sex, age, APOE4 status, tau, p-tau and baseline diagnosis*						
Trefoil factor 3^a^ (μg ml^−1^)					−0.21	0.007
N-terminal prohormone of brain natriuretic peptide^a^ (pg ml^−1^)					−0.16	0.05

Abbreviations: CSF, cerebrospinal fluid; EGF, endothelial growth factor; FDR, false discovery rate; p-tau, phosphorylated tau; t-tau, total tau.

a Transformed data. The statistics are presented for the transformed values (see Patients and Methods). Where data has been transformed, the units relate to data before transformation. None of the analytes in Supplementary Table 1 was FDR significant except those shown in this table.

Regression coefficients are shown for those measures showing FDR significant (5% level) associations.

*P*-values are FDR (5% level) corrected.

**Table 3:** Exploratory reverse stepwise regression analysis of CSF analytes with an FDR significant association with brain atrophy measurement in subjects with low CSF Aβ1-42 (⩽192 pg ml^−1^): when adjusted for t-tau and baseline volume; when adjusted for baseline volume, sex, age, APOE4 status, t-tau and p-tau; when adjusted for baseline volume, sex, age, APOE4 status, t-tau and p-tau and baseline diagnosis

*Brain atrophy*			*Ventricular expansion*			*Hippocampal atrophy*		
*Analyte*	*Adjusted regression coefficient*	P	*Analyte*	*Adjusted regression coefficient*	P	*Analyte*	*Adjusted regression coefficient*	P
*Adjusted for t-tau and baseline volume*								
TFF3^a^ (μg ml^−1^)	−12.3	0.001	TFF3^a^ (μg ml^−1^)	−4.7	<0.001	TFF3^a^ (μg ml^−1^)	−0.18	<0.001
Chromogranin-A (ng ml^−1^)	−0.04	0.006	Fibrinogen^a^ (mg ml^−1^)	−1.4	0.002			
			Angiotensin-converting enzyme^a^ (ng ml^−1^)	4.3	0.012			
			Macrophage colony-stimulating factor 1^a^ (ng ml^−1^)	−4.2	0.030			
			Chromogranin-A (ng ml^−1^)	−0.01	0.032			
								
*Adjusted for baseline volume, sex, age, APOE4 status, t-tau and p-tau*								
TFF3^a^ (μg ml^−1^)	−13.2	0.004	TFF3^a^ (μg ml^−1^)	−4.5	<0.001	TFF3^a^ (μg ml^−1^)	−0.23	<0.001
Chromogranin-A (ng ml^−1^)	−0.04	0.004						
								
*Adjusted for baseline volume, sex, age, APOE4 status, t-tau and p-tau and baseline diagnosis*								
						TFF3^a^ (μg ml^−1^)	−0.23	<0.001

Abbreviations: CSF, cerebrospinal fluid; FDR, false discovery rate; p-tau, phosphorylated tau; t-tau, total tau; TFF3, trefoil factor 3.

a Transformed data. The statistics are presented for the transformed values (see Patients and Methods). Where data has been transformed the units relate to data before transformation.

*P*-values shown here do not account for multiple comparisons.
