# Supplementary material for: Cerebrospinal fluid markers including trefoil factor 3 are associated with neurodegeneration in amyloid-positive individuals
Source: Transl Psychiatry. 2014 Jul 29;4(7):e419–. doi: 10.1038/tp.2014.58 (PMC4119225; doi:10.1038/tp.2014.58)
Supplement: Supplementary Table 1 [file tp201458x1.doc]

**Supplementary Table 1.**

Mean and standard deviation of each measured analyte across all 287 subjects.

| Cerebrospinal fluid analyte | Abbreviation | All Diagnoses (Mean ± SD) | Control (Mean ± SD) | MCI  (Mean ± SD) | AD  (Mean ± SD) |
| --- | --- | --- | --- | --- | --- |
| Alpha-1-Microglobulin (ug/mL)* | A1Micro | -1.3±0.22 | -1.4±0.22 | -1.3±0.22 | -1.3±0.23 |
| Alpha-2-Macroglobulin(mg/mL)* | A2Macro | -2.4±0.13 | -2.4±0.11 | -2.4±0.13 | -2.4±0.14 |
| Alpha-1-Antitrypsin(mg/mL)* | AAT | -2.3±0.15 | -2.4±0.15 | -2.3±0.14 | -2.4 0.17 |
| Angiotensin-Converting Enzyme(ng/mL)* | ACE | 0.3±0.16 | 0.3±0.16 | 0.3±0.15 | 0.3±0.16 |
| Adiponectin(ng/mL)* |  | -2.3±0.18 | -2.3±0.19 | -2.3±0.17 | -2.3±0.19 |
| Agouti Related Protein(pg/mL) | AGRP | 56.8±22.37 | 53.5±23.0 | 58.1±20.9 | 58.4±24.4 |
| Angiopoietin-2*(ng/mL) | ANG-2 | 0.01±0.17 | -0.001±0.16 | 0.03±0.18 | 0.002±0.18 |
| Apolipoprotein A-I*(mg/mL) | Apo A-I | -3.1±0.22 | -3.1±0.23 | -3.1±0.21 | -3.1±0.20 |
| Apolipoprotein C-III(ug/mL)* | Apo C-III | -1.2±0.22 | -1.2 ±0.3 | -1.2±0.21 | -1.3±0.21 |
| Apolipoprotein D(ug/mL)* | Apo D | 0.7±0.18 | 0.7±0.18 | 0.7±0.18 | 0.7±0.18 |
| Apolipoprotein E(ug/mL)* | Apo E | 0.8±0.14 | 0.8±0.13 | 0.8±0.14 | 0.8±0.15 |
| Apolipoprotein H(ug/mL)* | Apo H | -0.1±0.20 | -0.1±0.19 | -0.1±0.20 | -0.1±0.22 |
| AXL Receptor Tyrosine Kinase(ng/mL) | AXL | 4.2±1.42 | 4.2±1.37 | 4.2±1.5 | 3.9±1.0 |
| Beta-2-Microglobulin(ng/mL)* | B2M | 0.1±0.13 | 0.1±0.12 | 0.1±0.12 | 0.1±0.14 |
| Complement C3(mg/mL)* | C3 | -2.6±0.17 | -2.6±0.15 | -2.6±0.17 | -2.6±0.17 |
| Cancer Antigen 19-9(U/mL) | CA-19-9 | 1.1±0.50 | 1.2±0.5 | 1.2±0.5 | 1.0±0.4 |
| Calcitonin(pg/mL) * |  | 1.0±0.41 | 0.9±0.4 | 0.9±0.4 | 1.0±0.4 |
| CD 40 antigen(ng/mL)* | CD40 | -0.6±13 | -0.6±0.14 | -0.6±0.12 | -0.7±0.12 |
| Chromogranin-A(ng/mL) | CgA | 276.1±54.45 | 283.0±53.2 | 275.4±54.9 | 267.9±54.7 |
| Clusterin(ug/mL)* | CLU | 1.4±0.16 | 1.4±0.16 | 1.4±0.16 | 1.4±0.18 |
| Cortisol(ng/ml) |  | 16.1±5.93 | 15.6±6.3 | 16.5±5.5 | 16.0±6.4 |
| C-reactive protein(ug/mL)* | CRP | -2.8±0.56 | -2.7±0.5 | -2.9±0.5 | -2.9±0.7 |
| Cystatin-C(ng/mL)* |  | 0.4±0.084 | 0.4±0.08 | 0.4±0.09 | 0.4±0.08 |
| Endothelin-1(pg/mL) | ET1 | 12.4±3.69 | 12.3±4.1 | 12.7±3.6 | 12.0±3.4 |
| Fatty Acid-Binding Protein, heart(ng/mL)* | FABP, heart | 0.5±0.27 | 0.4±0.3 | 0.5±0.3 | 0.6±0.3 |
| Fas Ligand(pg/mL) | FasL | 11.2±4.81 | 10.9±4.2 | 11.6±5.5 | 10.8±3.9 |
| Fibroblast Growth Factor 4(pg/mL)* | FGF-4 | 1.6±0.15 | 1.6±0.16 | 1.7±0.14 | 1.7±0.15 |
| Fibrinogen(mg/mL)* |  | -3.4±0.38 | -3.5±0.4 | -3.4±0.4 | -3.3±0.3 |
| Ferritin(ng/ml)* | FRTN | 0.8±0.15 | 0.8±0.13 | 0.8±0.16 | 0.8±0.16 |
| Follicle-Stimulating Hormone(mIU/mL)* | FSH | -0.1±0.34 | -0.1±0.4 | -0.1±0.3 | -0.1±0.3 |
| Heparin-Binding EGF-Like Growth Factor(pg/mL)* | HB-EGF | 2.4±0.11 | 2.4±0.12 | 2.4±0.10 | 2.4±0.11 |
| Chemokine CC-4 (ng/ml)* | HCC-4 | -1.4±0.19 | -1.4±0.19 | -1.4±0.2 | -1.5±0.18 |
| Hepatocyte Growth Factor(ng/mL)* | HGF | 0.4±0.17 | 0.4±0.16 | 0.4±0.17 | 0.5±0.17 |
| T Lymphocyte-Secreted Protein I-309(pg/mL)* | I-309 | 1.3±0.17 | 1.3±0.17 | 1.3±0.16 | 1.3±0.20 |
| Intercellular Adhesion Molecule 1(ng/mL) | ICAM-1 | 1.0±0.44 | 0.9±0.4 | 1.0±0.5 | 1.0±0.4 |
| Immunoglobulin A(mg/mL)* | IgA | -2.5±0.31 | -2.5±0.3 | -2.5±0.3 | -2.5±0.3 |
| Interleukin-16(pg/mL)* | IL-16 | 0.9±0.19 | 0.9±0.19 | 0.9±0.18 | 0.9±0.18 |
| Interleukin-25(pg/mL) | IL-25 | 9.3±3.54 | 9.3±3.6 | 9.4±3.6 | 9.0±3.3 |
| Interleukin-3(ng/mL)* | IL-3 | -2.2±0.33 | -2.1±0.3 | -2.2±0.3 | -2.2±0.3 |
| Interleukin-6 receptor(ng/mL)* | IL-6r | -0.003±0.15 | 0.005±0.15 | -0.0008±0.15 | -0.02±0.14 |
| Interleukin-8(pg/mL)* | IL-8 | 1.7±0.14 | 1.7±0.13 | 1.7±0.16 | 1.7±0.13 |
| Insulin-like Growth Factor-Binding Protein(ng/mL) |  | 108.1±35.01 | 105.5 ±31.9 | 107.4 ±30.6 | 113.3±46.6 |
| Interferon gamma Induced Protein 10(pg/mL)* | IP-10 | 2.6±0.21 | 2.6±0.19 | 2.6±0.23 | 2.6±0.20 |
| Leptin(ng/mL)* | Leptin | -1.0±0.32 | -0.9±0.3 | -1.0±0.3 | -0.9±0.3 |
| Lectin-Like Oxidized LDL Receptor 1(ng/mL) | LOX-1 | 6.4±2.24 | 6.2±2.1 | 6.5±2.3 | 6.4±2.3 |
| Apolipoprotein(ug/mL) * | LP(a) | -1.6±0.52 | -1.6±0.6 | -1.6±0.5 | -1.5±0.6 |
| Monocyte Chemotactic Protein 1(pg/mL)* | MCP-1 | 2.7±0.13 | 2.7±0.12 | 2.7±0.13 | 2.7±0.14 |
| Monocyte Chemotactic Protein 2(pg/mL)* | MCP-2 | 0.5±0.17 | 0.6±0.17 | 0.5±0.18 | 0.5±0.16 |
| Macrophage Colony-Stimulating Factor 1(ng/mL)* | M-CSF | -0.2±0.14 | -0.2±0.14 | -0.2±0.14 | -0.2±0.13 |
| Macrophage Migration Inhibitory Factor(ng/mL)* | MIF | -0.7±0.33 | -0.7±0.3 | -0.6±0.3 | -0.6±0.3 |
| Monokine Induced by Gamma Interferon(pg/mL)* | MIG | 2.4±0.32 | 2.4±0.3 | 2.4±0.3 | 2.4±0.3 |
| Macrophage Inflammatory Protein-1 beta(pg/mL)* | MIP-1 beta | 1.2±0.19 | 1.2±0.21 | 1.2±0.20 | 1.2±0.15 |
| Matrix Metalloproteinase-2(ng/mL)* | MMP-2 | 0.9±0.23 | 0.9±0.22 | 0.9±0.25 | 0.8±0.23 |
| Matrix Metalloproteinase-3(ng/mL)* | MMP-3 | -0.5±0.20 | -0.5±0.18 | -0.5±0.20 | -0.4±0.19 |
| Myoglobin(ng/mL)* |  | -0.4±0.4 | -0.4±0.4 | -.0.4±0.4 | -0.4±0.5 |
| Neutrophil Gelatinase-Associated Lipocalin(ng/mL)* | NGAL | 0.2±0.20 | 0.2±0.20 | 0.3±0.21 | 0.3±0.18 |
| N-terminal prohormone of brain natriuretic peptide(pg/mL)* | NT proBNP | 2.2±0.15 | 2.1±0.15 | 2.2±0.15 | 2.2±0.15 |
| Osteopontin(ng/mL) |  | 33.4±9.8 | 31.4±10.0 | 33.9±9.7 | 35.1±9.0 |
| Plasminogen Activator Inhibitor 1(ng/mL)* | PAI-1 | -0.02±0.17 | -0.03±0.16 | -0.02±0.17 | 0.02±0.19 |
| Prostatic Acid Phosphatase(ng/mL)* | PAP | -0.8±0.19 | -0.8±0.21 | -0.8 0.18 | -0.8±0.18 |
| Pregnancy-Associated Plasma Protein A (mIU/mL)* | PAPP-A | -2.0± 0.17 | -2.0±0.14 | -2.0±0.19 | -2.0±0.17 |
| Placenta Growth Factor(pg/mL)* | PLGF | 1.8±0.20 | 1.8±0.21 | 1.8±0.20 | 1.8±0.19 |
| Pancreatic Polypeptide(pg/mL)* | PPP | 0.4±0.32 | 0.3±0.3 | 0.4±0.3 | 0.5±0.3 |
| Prolactin(ng/mL)* | PRL | 0.3±0.13 | 0.2±0.11 | 0.3±0.13 | 0.3±0.15 |
| T-Cell-Specific Protein RANTES(ng/mL)* | RANTES | -2.6±0.3 | -2.5±0.4 | -2.6±0.24 | -2.6±0.24 |
| Resistin(ng/mL)* | Resistin | -1.4±0.3 | -1.4±0.3 | -1.4±0.3 | -1.3±0.3 |
| S100 calcium-binding protein B(ng/mL)* | S100-B | 0.4±0.15 | 0.4±0.2 | 0.4±0.16 | 0.5±0.14 |
| Serum Amyloid P-Component(ug/mL)* | SAP | -2.6±0.3 | -2.6±0.3 | -2.6±0.3 | -2.6±0.3 |
| Stem Cell Factor(pg/mL)* | SCF | 1.6±0.17 | 1.6±0.16 | 1.6±0.17 | 1.6±0.15 |
| Serum Glutamic Oxaloacetic Transaminase(ug/mL)* | SGOT | 0.6±0.11 | 0.6±0.12 | 0.6±0.10 | 0.6±0.11 |
| Sex Hormone-Binding Globulin(nmol/L)* | SHBG | -0.9±0.24 | -0.9±0.23 | -0.9±0.24 | -0.9±0.24 |
| Sortilin(ng/mL)* | Sortilin | 0.8±0.11 | 0.8±0.10 | 0.8±0.11 | 0.8±0.11 |
| Thyroxine-Binding Globulin(ug/mL)* | TBG | -0.7±0.21 | -0.7±0.18 | -0.7±0.22 | -0.6±0.23 |
| Tissue Factor(ug/mL)* | TF | 0.6±0.21 | 0.5±0.20 | 0.6±0.21 | 0.5±0.20 |
| Trefoil Factor 3(ug/mL)* | TFF3 | -1.8±0.18 | -1.7±0.18 | -1.8±0.17 | -1.8±0.20 |
| Transforming Growth Factor alpha(pg/mL)* | TGF-alpha | 1.0±0.28 | 1.1±0.23 | 1.0±0.3 | 1.0±0.3 |
| Tissue Inhibitor of Metalloproteinases 1(ng/mL)* | TIMP-1 | 1.6±0.13 | 1.6±0.10 | 1.6±0.13 | 1.6±0.16 |
| Thrombomodulin (ng/mL)* | TM | -0.9±0.16 | -0.9±0.17 | -0.9±0.17 | -0.9±0.14 |
| Tumor Necrosis Factor Receptor 2 (ng/mL)* | TNFR2 | -0.1±0.15 | -0.2±0.14 | -0.1±0.15 | -0.1±0.16 |
| TNF-Related Apoptosis-Inducing Ligand Receptor 3 (ng/mL)* | TRAIL-R3 | -0.2±0.16 | -0.2±0.14 | -0.2±0.16 | -0.2±0.16 |
| Vascular Cell Adhesion Molecule-1 (ng/mL)* | VCAM-1 | 1.2±0.14 | 1.1±0.12 | 1.2±0.15 | 1.2±0.15 |
| Vascular Endothelial Growth Factor (pg/mL)* | VEGF | 2.7±0.13 | 2.7±0.12 | 2.7±0.13 | 2.7±0.13 |
| von Willebrand Factor(ug/mL)* | vWF | -1.5±0.17 | -1.4±0.17 | -1.5±0.16 | -1.5±0.17 |

*: transformed data. The statistics are presented for the transformed values (see methods). Where data has been transformed the units relate to data before transformation.
